# Internet of things-based pulmonary rehabilitation for moderate-to-severe chronic obstructive pulmonary disease: a prospective non-randomized controlled intervention study protocol

**DOI:** 10.3389/fmed.2026.1861226

**Published:** 2026-06-09

**Authors:** Nianci Guo, Shukun Chai, Runlu Wang, Xiaoqian Gu, Jie Chen, Huikun Zhao, Kaili Qie, Wentao Ni, Jinying Shi

**Affiliations:** 1 Department of Pulmonary and Critical Care Medicine, Shijiazhuang People’s Hospital, Shijiazhuang, Hebei, China; 2 Department of Pulmonary and Critical Care Medicine, Peking University People’s Hospital, Beijing, China; 3 Department of Pulmonary and Critical Care Medicine, Peking University People's Hospital Shijiazhuang Hospital, Shijiazhuang, Hebei, China

**Keywords:** acute exacerbation, chronic obstructive pulmonary disease, internet of things, pulmonary rehabilitation, study protocol

## Abstract

Pulmonary rehabilitation (PR) constitutes a cornerstone of non-pharmacological management in chronic obstructive pulmonary disease (COPD); however, its clinical effectiveness is frequently attenuated by suboptimal patient adherence and the absence of individualized, continuous monitoring within the home setting. Internet of Things (IoT) technology offers a promising solution to these persistent challenges; however, comprehensive closed-loop management models that fully integrate IoT capabilities remain underexplored. This prospective, non-randomized controlled intervention study will enroll 588 patients with moderate-to-severe stable COPD (GOLD grades 2–4) from a single institution. Participants will be assigned to either conventional PR (Group A) or IoT-assisted PR (Group B) based on initial preference and technology readiness. Group B will receive IoT-enabled devices and a smartphone app to support home-based PR, including daily respiratory muscle training and structured aerobic/resistance exercise (3–5 sessions/week). Group A will receive standard PR education and guidance. Follow-ups will occur at weeks 4, 12, 26, and 52. The primary outcome is the 12-month rate of moderate-to-severe AECOPD. Secondary outcomes include pulmonary function, exercise capacity, HRQoL, right cardiac function, pneumonia incidence, all-cause mortality, and adherence. Analyses will be performed on an intention-to-treat basis. This study will evaluate the efficacy of an IoT-based pulmonary rehabilitation management model in reducing AECOPD-related readmissions and improving exercise tolerance, quality of life, and dyspnea in patients with moderate-to-severe COPD, while examining its feasibility and safety in a real-world setting. The findings may support the development of a technology-enabled continuous care model for chronic respiratory diseases, facilitating the transition from hospital-centric to patient-centric care and advancing the application of smart nursing and remote health management. The study protocol has been registered with the Chinese Clinical Trial Registry (ChiCTR2500106412).

## 1 Introduction

Chronic obstructive pulmonary disease (COPD) is a common, preventable, and treatable condition characterized by persistent respiratory symptoms and airflow limitation, imposing a substantial and escalating burden on healthcare systems globally (1, 2). In China, the prevalence among adults aged 40 years and older has been reported at 13.7%, establishing COPD as a primary public health priority (1). The chronic and progressive nature of COPD, coupled with recurrent acute exacerbations (AECOPD), drives progressive declines in exercise capacity and health-related quality of life (HRQoL), as well as substantially increased healthcare costs (2).

Although pharmacological therapy remains indispensable, non-pharmacological interventions—particularly pulmonary rehabilitation—are equally critical for optimizing patient outcomes (1). Pulmonary rehabilitation (PR) is defined as a comprehensive, individualized intervention that includes exercise training, health education, and behavior modification (3). This intervention aims to improve the physical and psychological condition of individuals with chronic respiratory diseases and to promote sustained adherence to health-enhancing behaviors (4, 5). Robust evidence has confirmed that PR confers clinically meaningful improvements in dyspnea, exercise capacity, and quality of life in patients with COPD (6–9). Accordingly, the Global Initiative for Chronic Obstructive Lung Disease (GOLD) strongly recommends PR for patients with moderate-to-severe disease (10).

Despite the well-established efficacy of PR, its implementation in traditional home settings without remote clinician supervision often yields suboptimal adherence and clinical benefits due to lack of real-time monitoring and individualized feedback (11, 12). Patients frequently lack the knowledge, motivation, and ongoing support needed to maintain complex exercise regimens, leading to attenuated long-term benefits (13, 14). Key barriers include insufficient awareness of the importance of PR, absence of real-time professional guidance, and a lack of individualized, engaging rehabilitation programs. Accordingly, developing innovative, patient-centered models that enhance the accessibility, engagement, and personalization of home-based PR represents an urgent clinical need (11, 15).

Internet of Things (IoT) technology, enabling real-time data exchange between patients and healthcare providers through interconnected devices and platforms, represents a significant advancement in contemporary healthcare delivery (16). Although IoT has been explored for continuous vital sign monitoring in COPD management (17–20), existing evidence for PR is limited, especially for patients with moderate-to-severe COPD and a history of exacerbations. Most studies are characterized by small sample sizes and short follow-up periods, precluding robust assessment of long-term clinical outcomes. Moreover, substantial heterogeneity in intervention design has hindered the development of a standardized IoT-based PR management model (21).

In response to these gaps, the present study aims to conduct a prospective cohort investigation to develop and evaluate an IoT-based PR management model and to assess its impact on clinical outcomes in patients with moderate-to-severe COPD. The specific objectives are as follows: (1) to compare the effect of an IoT-assisted PR management model versus conventional care on the rate of AECOPD-related readmissions within 12 months post-discharge; (2) to evaluate the model’s efficacy in improving exercise tolerance, dyspnea, HRQoL, pulmonary function, and rehabilitation adherence; and (3) to examine the safety and feasibility of IoT-assisted PR management, thereby providing evidence-based support for clinical practice.

## 2 Methods and analysis

### 2.1 Study design

**Figure 1:**
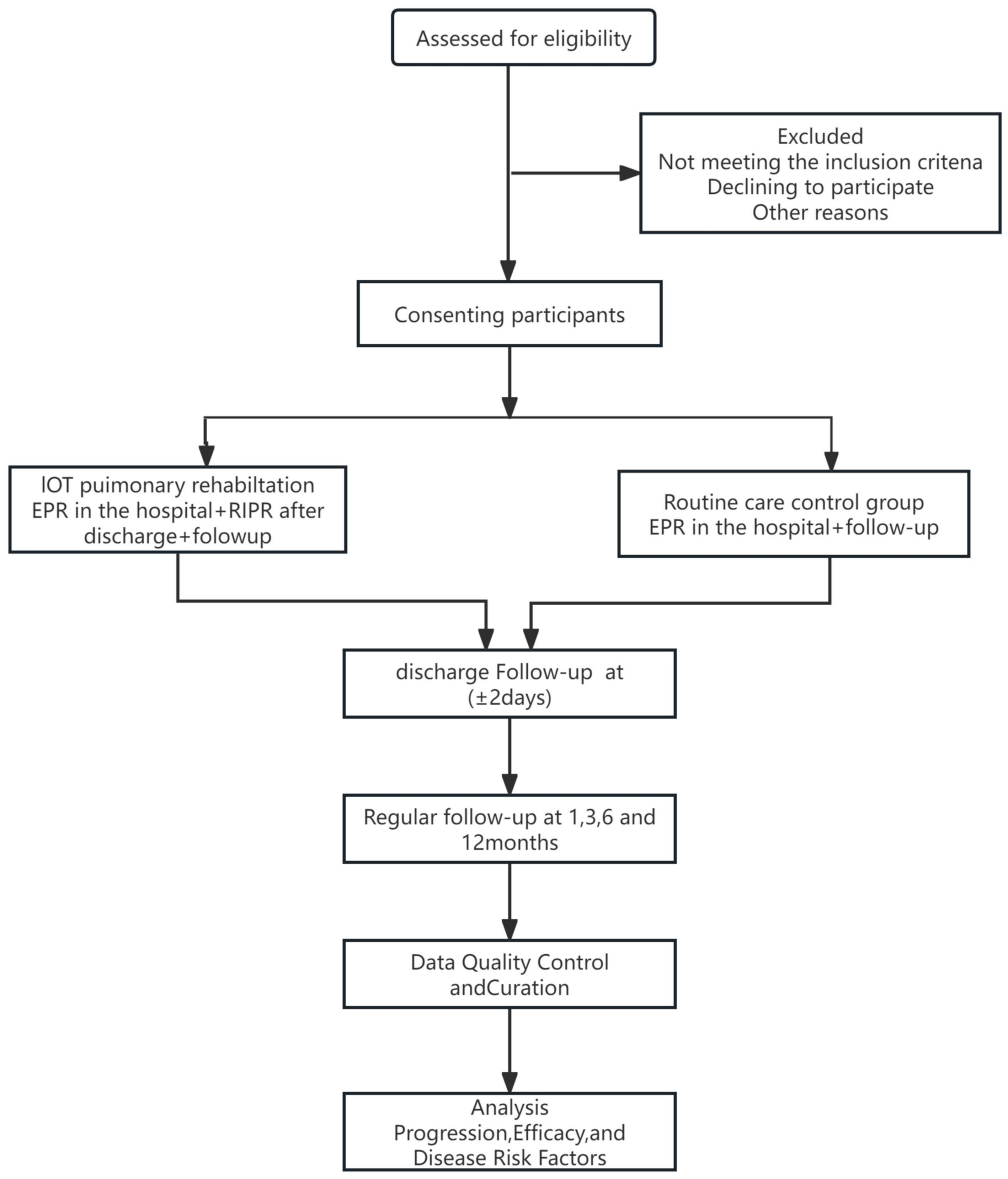
Flow chart of the study.

This is a prospective, pragmatic, non-randomized controlled intervention study with a 52-week follow-up period. The intervention is actively assigned based on patient preference and technology readiness, and the study includes a concurrent control group receiving standard care. The study protocol has been registered with the Chinese Clinical Trial Registry (ChiCTR2500106412). Study design and reporting will adhere to the Standard Protocol Items: Recommendations for Interventional Trials (SPIRIT) guidelines (22). Figure 1 is the trial flow chart.

### 2.2 Study setting and participants

Participants will be recruited from the Department of Respiratory and Critical Care Medicine, Shijiazhuang People’s Hospital, Shijiazhuang, Hebei Province, China.

#### 2.2.1 Inclusion criteria

1. Age between 40 and 75 years.
2. A confirmed diagnosis of COPD according to GOLD criteria, defined as a post-bronchodilator FEV₁/FVC ratio < 0.70 and FEV₁ % predicted < 80% (GOLD grades 2–4) at enrollment.
3. A history of at least two moderate acute exacerbations or one severe acute exacerbation requiring hospitalization within the preceding 12 months (Group E according to the GOLD guideline).
4. Clinical stability for a minimum of four weeks prior to enrollment, defined as the absence of acute exacerbations or changes in pharmacological regimen.
5. Capacity to provide written informed consent and willingness to comply with all study procedures, including utilization of IoT devices and the smartphone application.

#### 2.2.2 Exclusion criteria

1. Inability to participate in PR or operate IoT devices due to severe cognitive impairment, physical disability, or communication barriers.
2. Presence of other significant respiratory comorbidities (e.g., active pulmonary tuberculosis, lung malignancy, or bronchiectasis as the primary diagnosis), unstable cardiac conditions (e.g., recent myocardial infarction), or other comorbidities that would preclude safe participation in PR.
3. Current enrollment in another interventional clinical trial.
4. Pregnancy or lactation.

#### 2.2.3 Study withdrawal criteria

Participants meeting any of the following criteria will be withdrawn from the study following enrollment:

1. Development of a concurrent acute illness requiring intervention that would confound study outcomes.
2. Failure to complete required assessments, rendering evaluation of the primary outcome impossible.
3. Occurrence of a serious adverse event during follow-up that, in the investigator’s judgment, necessitates withdrawal from the trial.
4. Pregnancy occurring during the follow-up period.
5. Voluntary withdrawal, loss to follow-up, or death during the follow-up period.
6. Post-enrollment discovery that the participant does not fulfill any inclusion criterion or meets any exclusion criterion.

### 2.3 Sample size calculation

The primary outcome measure is the 12-month rate of moderate-to-severe AECOPD. Based on prior literature, the annual exacerbation frequency in patients with moderate-to-severe COPD ranges from 0.5 to 3.0 events, of which approximately 60% are classified as moderate or severe. Assuming a two-sided significance level (*α*) of 0.05 and a statistical power (1 − *β*) of 0.80, and informed by preliminary data and published evidence suggesting that PR interventions reduce exacerbation frequency by approximately 20% (risk ratio [RR] = 0.80), sample size calculations performed using PASS 2023 software indicated a requirement of 267 participants per group. Accounting for an estimated attrition rate of 10%, which is considered feasible given the chronic disease management context in the Shijiazhuang region, a 10% inflation factor was applied, yielding a final required sample size of 294 participants per group and a total sample of 588 participants.

### 2.4 Participant recruitment and group allocation

Eligible patients will be identified and recruited by clinicians within the Department of Respiratory and Critical Care Medicine at Shijiazhuang People’s Hospital. Research nurses will provide prospective participants with comprehensive information regarding the study. Given the inherent nature of the intervention—which necessitates active patient engagement with technology—strict randomization may compromise adherence and ecological validity. Therefore, following the provision of written informed consent, participants will be allocated to either the IoT-assisted PR group or the conventional PR group based on their initial preference and a preliminary assessment of technology readiness and digital competency (e.g., smartphone ownership, basic digital literacy). This pragmatic allocation approach is designed to reflect real-world implementation conditions. Participants who initially prefer conventional care but express willingness to engage with IoT technology will be encouraged to join the IoT-assisted PR group to maximize recruitment efficiency. Baseline characteristics of both groups will be rigorously compared and statistically adjusted in all subsequent analyses. We acknowledge that an attention-matched control group is not feasible in this pragmatic study design due to resource constraints and the inherent nature of the IoT intervention. This limitation will be discussed more clearly in the Discussion section.

### 2.5 Interventions

**Figure 2:**
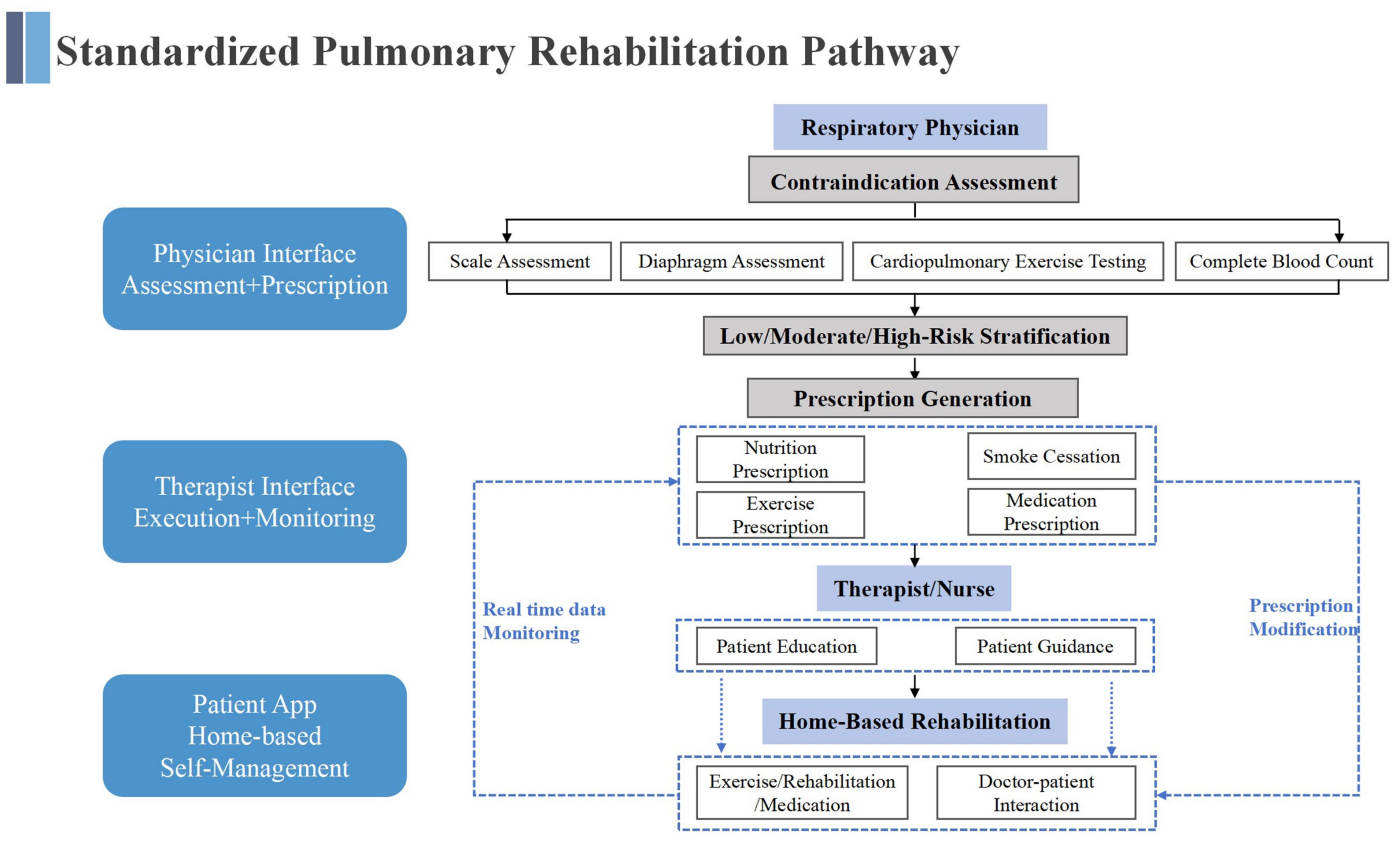
Standard pulmonary rehabilitation pathway. The conventional pulmonary rehabilitation group (control group) will receive physician interface assessment and therapist interface execution in hospital; the IoT-enhanced pulmonary rehabilitation group (intervention group) will receive a comprehensive IoT-based rehabilitation management, in addition to physician interface assessment and therapist interface execution in hospital.

Figure 2 illustrates the pulmonary rehabilitation pathway in this study.

#### 2.5.1 Conventional pulmonary rehabilitation group (control group)

Participants will receive standard care consistent with GOLD guidelines, comprising the following components:

##### 2.5.1.1 In-hospital education

A structured educational program delivered by nurses and physiotherapists, covering COPD pathophysiology, pharmacological management (including inhaler technique), energy conservation strategies, nutritional guidance, and smoking cessation counseling.

##### 2.5.1.2 Standardized home-based PR prescription at discharge

Participants will receive verbal and written instructions for home-based PR, including:

- Breathing exercises: pursed-lip breathing and diaphragmatic breathing, 15 min per session, twice daily.
- Resistance training: upper limb strengthening exercises using standardized resistance bands (e.g., lateral arm raises at 40–50% of one-repetition maximum), three sets of ten repetitions per day.

##### 2.5.1.3 Follow-up assessments

Outpatient clinic visits will be scheduled at 1, 3, 6 and 12 months for clinical evaluation, spirometry, and questionnaire administration. No remote monitoring or proactive feedback regarding PR adherence will be provided during the follow-up period.

#### 2.5.2 IoT-enhanced pulmonary rehabilitation group (intervention group)

**Figure 3:**
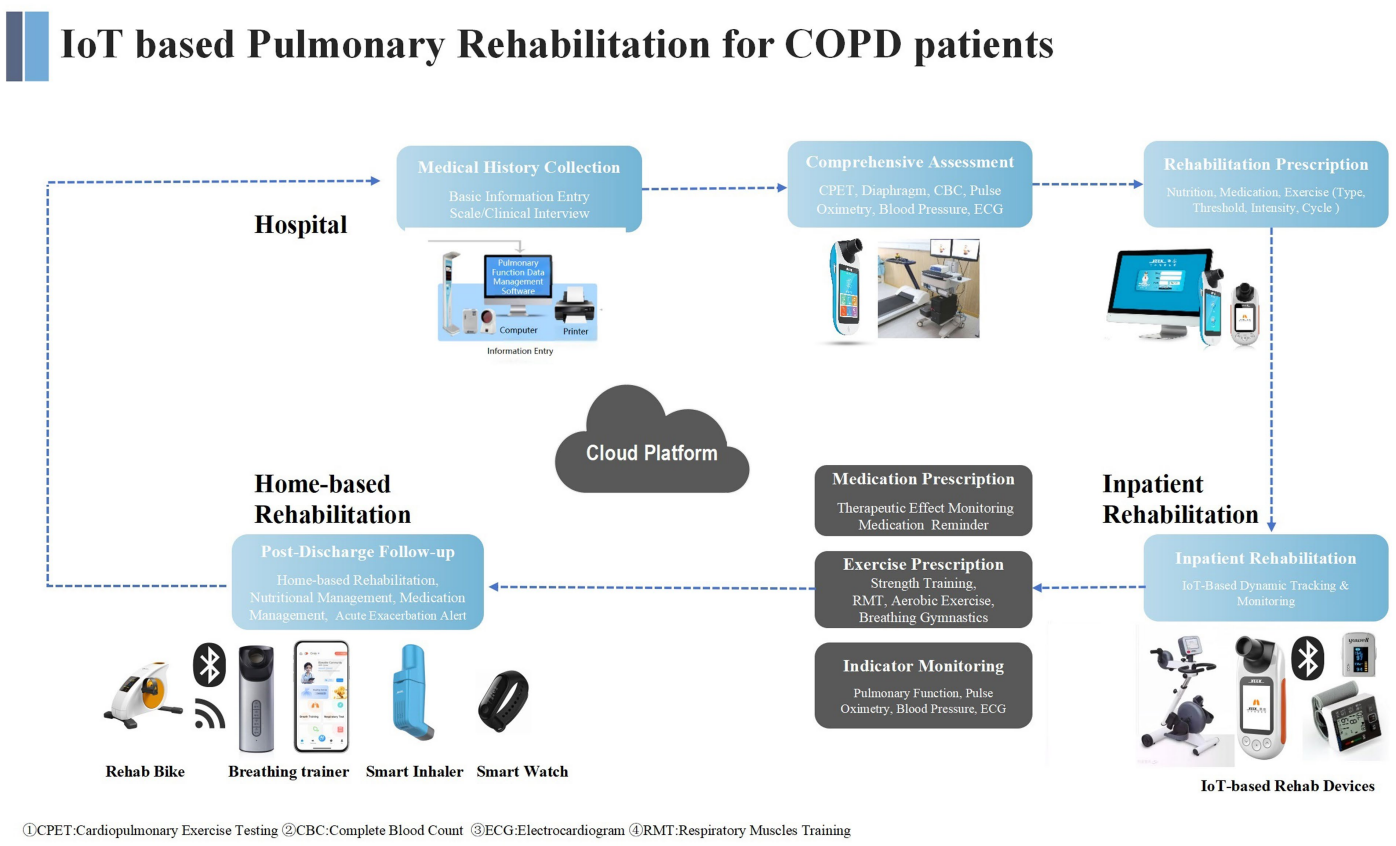
Architecture of the IoT-based pulmonary rehabilitation management platform, illustrating the closed-loop interaction among patients, devices, and the clinical team.

In addition to standard education and the initial PR prescription, participants in this group will receive a comprehensive IoT-based management model. The system architecture of IoT-based platform is illustrated in Figure 3.

1. IoT platform and devices: participants will be provided with an IoT-enabled threshold-load respiratory training device and a dedicated smartphone application (“Breathing Tribe,” patient-facing interface). A clinician portal and therapist application will facilitate remote management. A pulse oximeter will be provided to ensure exercise safety monitoring.
2. Individualized prescription delivery via the platform: at discharge, clinicians will use the portal to generate personalized, dynamic PR prescriptions based on baseline assessment findings (e.g., 6MWT results, MIP), which will be electronically transmitted to the patient’s application. The prescription will encompass:

- Respiratory muscle training (RMT): twice daily using the IoT device. Initial intensity will be set at 30% of MIP, with weekly increments of 5%, up to a maximum of 60% MIP. The device will automatically record training frequency, duration, and achieved inspiratory/expiratory pressures.
- Aerobic and resistance training: home-based walking prescribed at 50–60% of the average speed achieved during the 6MWT, combined with resistance band exercises (identical protocol to the control group). The application will provide instructional videos and a built-in timer.
- Medication and nutritional management: the application will incorporate medication reminders and deliver personalized dietary recommendations based on Nutritional Risk Screening 2002 (NRS2002) assessment results.

The following specifications are added to the intervention protocol

- Exact training duration: each RMT session will last approximately 15–20 min. Each aerobic training session (walking) will last 20–30 min per session, with resistance training lasting an additional 10–15 min.
- Target weekly exercise time: total target weekly exercise time is 150–180 min, distributed as 3–5 sessions per week (approximately 30–50 min per session).
- Progression rules for walking training: based on patient-reported tolerance and the absence of adverse events, advancement is carried out after three consecutive training sessions have been completed. Each increase is 5 min, until reaching 30–45 min. Then the intensity is adjusted, with walking speed increased by approximately 0.5 km/h each time. At least two weeks of observation are required after each adjustment. If tolerated, the target walking speed will be increased from 50–60% of the average speed on the 6-min walk test to 60–70% after 4 weeks.
- Progression rules for resistance training: when participants can comfortably complete 2 sets of 12 repetitions each, the number of repetitions for the resistance band training will be increased, up to a maximum of 15 repetitions. After 2 weeks of observation, if there are no adverse reactions, the intensity (differentiated by color-coded resistance levels) will be increased to the next resistance level, maintaining the same repetition target. Once both the number of repetitions and the resistance level have been successfully increased, the number of sets will be increased from 2 sets to 3 sets. The increase in the number of sets must only be carried out after the intensity and number of repetitions have been stabilized.
- Stopping criteria during exercise: participants will be instructed to stop exercise immediately if they experience chest pain, severe dyspnea (mMRC ≥ 3), dizziness, palpitations, or intolerable fatigue.
- Safety thresholds for SpO₂ or symptoms: exercise will be paused if SpO₂ drops below 88% or falls by ≥4% from baseline resting saturation. Exercise may resume when SpO₂ returns to ≥90% and symptoms resolve. If SpO₂ remains below 88% after two minutes of rest, the session should be terminated and the clinician notified. For symptoms, exercise will be stopped if Borg dyspnea score ≥ 5 or Borg fatigue score ≥ 5 (on 0–10 scale).

## 3 Remote monitoring, feedback, and dynamic prescription adjustment

- Real-time data transmission: IoT devices will automatically transmit RMT data to the platform. Participants will log aerobic and resistance training completion via the application.
- Automated feedback and alert system: the application will deliver daily reminders, motivational messages, and visualized progress charts. Automated alerts will be generated and transmitted to the responsible therapist if a participant misses scheduled training sessions or if physiological parameters (e.g., SpO₂) fall below predefined thresholds.
- Clinician-led monitoring and dynamic adjustment: respiratory therapists will remotely review patient adherence and performance data on a weekly basis via the therapist dashboard. Feedback will be communicated through in-application messaging or telephone. In collaboration with supervising physicians, therapists will dynamically adjust PR prescriptions through the platform as clinically indicated (e.g., increasing RMT resistance, modifying aerobic targets), thereby establishing a continuous closed-loop management cycle.

### 3.1 Outcome measures and assessment instruments

#### 3.1.1 Primary outcome

##### 3.1.1.1 The primary outcome is the 12-month rate of moderate-to-severe AECOPD

Defined as the number of moderate or severe acute exacerbation events occurring per patient during the 12-month follow-up period. AECOPD is defined as an acute deterioration of respiratory symptoms (dyspnea, cough, and/or sputum production) requiring treatment with systemic corticosteroids and/or antibiotics, or necessitating hospitalization (10). Exacerbation data will be ascertained through review of the hospital information system and confirmed via structured telephone follow-up.

#### 3.1.2 Secondary outcomes

1. Exercise tolerance: assessed using the six-minute walk distance (6MWD), conducted in accordance with American Thoracic Society (ATS) guidelines along a 30-meter flat corridor. Measurements will be obtained at baseline and at 3, 6, and 12 months post-intervention.
2. Dyspnea severity: evaluated using the modified British Medical Research Council (mMRC) dyspnea scale, a single-item, five-level ordinal scale (scored 0–4), with higher scores indicating greater dyspnea severity.
3. Health-related quality of life: assessed jointly using the COPD Assessment Test (CAT) and the St. George’s Respiratory Questionnaire (SGRQ). The CAT comprises 8 items with a total score ranging from 0 to 40, where higher scores indicate worse HRQoL. The SGRQ comprises 50 items encompassing symptoms, activity limitation, and disease impact domains, with a total score ranging from 0 to 100, where higher scores indicate worse HRQoL.
4. Pulmonary function: measured using a portable spirometer, capturing FEV₁ % predicted, FVC, and peak expiratory flow (PEF). All measurements will be performed by trained research nurses according to standardized operating procedures.
5. Rehabilitation adherence: in the intervention group, adherence will be assessed using the following metrics: (i) *device utilization adherence rate*, defined as daily data upload completion rate ≥ 80%; (ii) *training completion rate*, calculated as the number of days on which prescribed rehabilitation was completed divided by the total prescribed training days, multiplied by 100%; and (iii) *follow-up completion rate*, defined as the proportion of participants completing assessments at each scheduled timepoint.
6. Adverse events: all adverse events occurring during the study period will be systematically recorded, including but not limited to falls, fractures, or cardiovascular events associated with rehabilitation training; disease deterioration events; and unplanned healthcare utilization events.

### 3.2 Covariates

Baseline data will be systematically collected, encompassing: demographic characteristics (age, sex, educational attainment, living arrangements, and health insurance type); disease characteristics (COPD duration, GOLD grade, prior exacerbation history, and comorbidity burden quantified using the Charlson Comorbidity Index); smoking history; current pharmacological regimen; and baseline pulmonary function parameters. We acknowledge that allocation based on patient preference and technology readiness may introduce selection bias. Patients who are more comfortable with smartphones and IoT devices may also be younger, more educated, more motivated, more adherent, or have better social support. All of these factors could influence the outcomes. Therefore, the following additional variables will be collected to allow for statistical adjustment: digital literacy, baseline motivation, and socioeconomic status.

### 3.3 Data collection and follow-up procedures

Dedicated research nurses will be appointed to oversee participant recruitment, baseline assessment, follow-up coordination, and data entry. Data will be collected and managed using the FREE Electronic Data Capture System (version 2.0; Beijing FreeClinical Medical Technology Co., Ltd., Beijing, China). All outcome assessors will be blinded to group allocation throughout the follow-up phase to the extent feasible. The schedule of assessments across follow-up timepoints is presented in Supplementary Tables S1, S2.

### 3.4 Statistical analysis

Data will be analyzed using SPSS version 23.0 and R (version 4.2 or above). The following analytical approaches will be employed:

(1) Data description: continuous variables will be assessed for normality using appropriate statistical tests. Normally distributed data will be expressed as mean ± standard deviation (x̄ ± s); non-normally distributed data will be presented as median (interquartile range). Categorical variables will be summarized as frequencies (*n*) and proportions (%).
(2) Between-group comparisons: categorical variables will be compared using the chi-square test or Fisher’s exact test, as appropriate. Normally distributed continuous variables will be analyzed using the independent samples t-test; within-group pre-post comparisons will employ the paired t-test. Non-normally distributed variables will be analyzed using non-parametric equivalents (Mann–Whitney U test or Wilcoxon signed-rank test). Multiple group comparisons will utilize one-way analysis of variance (ANOVA) with appropriate post-hoc correction (LSD or Bonferroni). Longitudinal data across repeated time points will be analyzed using repeated measures ANOVA or generalized estimating equations (GEE).
(3) Multivariable analysis: to delineate the independent association between the IoT intervention and primary outcome measures (e.g., pulmonary function improvement, AECOPD frequency, readmission rate), multivariable logistic regression or Cox proportional hazards models will be employed, with adjustment for pre-specified potential confounders (including age, sex, comorbidities, and smoking history).
(4) Missing data: missing data for key variables will be addressed using single and multiple imputation techniques to preserve sample integrity and maximize analytical efficiency.
(5) Significance threshold: all statistical tests will be two-sided, with a significance level of *p* < 0.05. Where applicable, multiple testing corrections (e.g., Bonferroni correction) will be implemented to control the family-wise type I error rate.

### 3.5 Ethical considerations

This study has received approval from the Institutional Ethics Committee of Shijiazhuang People’s Hospital (Approval No.: ChiCTR2500106412) and will be conducted in full accordance with the principles of the Declaration of Helsinki and applicable Chinese regulatory requirements. Written informed consent will be obtained from all participants prior to the initiation of any study-related procedures. Participants will be explicitly informed of their right to withdraw from the study at any time without consequence. All data will be anonymized and stored in a secure, confidential manner.

## 4 Discussion

This study has designed an IoT-based pulmonary rehabilitation management model for patients with moderate-to-severe COPD and aims to validate its clinical effectiveness through a prospective cohort design. The study addresses a fundamental challenge in COPD management. Patients with moderate-to-severe disease and exacerbation history represent the highest-risk population in COPD, yet traditional PR models are constrained by geographic inaccessibility, limited resource availability, and insufficient patient motivation, thereby failing to reach those most in need of rehabilitation (15, 23). By transcending the temporal and spatial limitations of center-based care, IoT-assisted PR extends professional rehabilitation guidance into patients’ homes, with the potential to substantially enhance PR coverage rates and adherence.

The principal innovations of this study are as follows. First, the study establishes a standardized IoT-assisted PR nursing management protocol with clearly delineated operational procedures and nursing responsibilities, ensuring reproducibility and scalability. Second, a tiered early-warning response mechanism is introduced, transforming passive follow-up into proactive monitoring and timely intervention, thereby enhancing the efficiency and precision of risk management. Third, behavioral incentivization strategies (including a point-based reward system) are integrated into rehabilitation management to specifically address the persistent challenge of long-term adherence. Fourth, a mixed-methods evaluation framework is adopted—combining quantitative outcome assessment with process indicators—to address not only whether the intervention is effective, but to elucidate the mechanisms through which it achieves its effects.

It should be noted that the intervention model developed in our study embeds explicit nursing science content. Research nurses within the IoT management framework fulfill multiple, complementary roles (monitor, educator, coordinator, and early warning responder) through daily data review, risk identification, telephone follow-up, rehabilitation guidance, and multidisciplinary resource coordination. These functions represent the core elements of continuity of care and chronic disease management (24). The findings will therefore provide novel operational models and evidence-based support for specialized nursing practice in chronic respiratory disease.

Several potential challenges and corresponding mitigation strategies have been considered when performing the study. First, to address device usability issues and the digital divide among elderly patients, one-on-one training will be provided at enrollment, supplemented by illustrated user manuals, a dedicated technical support helpline, and reserve equipment. Second, data privacy and security concerns will be managed through comprehensive informed consent, enterprise-grade encryption and de-identification protocols, compliance with applicable cybersecurity legislation, and regular security audits. Third, to sustain long-term adherence, a multidimensional approach will be implemented, including personalized rehabilitation progress reports, peer support networks, regular proactive telephone follow-up, and timely outreach to participants with no data upload for three consecutive days. Fourth, to mitigate potential confounding bias inherent in the non-randomized design, a comprehensive set of covariates—including disease severity, comorbidity burden, and socioeconomic status—will be collected and adjusted for using multivariable regression and propensity score analyses, with sensitivity analyses conducted to evaluate the robustness of the primary outcome findings. Finally, we acknowledge that an attention-matched control group is not feasible in this pragmatic study design due to resource constraints and the inherent nature of the IoT intervention. Digital literacy, access to technology, acceptability, and user-centric design may significantly influence both group assignment and outcomes. As highlighted by Mishra et al. in their systematic review on digital interventions for COPD (25), factors such as usability, user-centric design, technology acceptance and adoption play critical roles in the effectiveness of digital interventions. Patients with higher digital literacy and better access to technology may be more likely to be assigned to or accept the IoT intervention, and may also achieve better outcomes regardless of the intervention itself. These factors will be measured at baseline and adjusted for in the primary analyses.

Our study has several limitations. First, the non-randomized design offers a lower level of evidence than a randomized controlled trial and is susceptible to unmeasured confounding. Second, the nature of the intervention precludes blinding of participants and intervention providers, which may introduce performance and detection bias, although outcome assessors will be blinded where feasible. Third, the 12-month follow-up may be insufficient to assess long-term outcomes. Fourth, the absence of a formal health economic analysis precludes a comprehensive assessment of the intervention’s cost–benefit profile. In the future, we will perform multicenter randomized controlled trials to address these limitations.

In summary, this study protocol delineates a prospective cohort investigation of an IoT-based pulmonary rehabilitation management model for patients with moderate-to-severe COPD. The study aims to validate the efficacy of IoT-assisted PR in reducing AECOPD-related readmission rates and improving exercise tolerance, health-related quality of life, and dyspnea, while examining its real-world feasibility and safety. The anticipated findings are expected to provide evidence-based support for establishing a nurse-led, technology-enabled model of continuous care for chronic respiratory diseases, thereby facilitating the transition from a hospital-centric to a patient-centric care paradigm and advancing the practical application of smart nursing and remote health management in the field of chronic respiratory diseases.
