# Supplementary material for: Internet of things-based pulmonary rehabilitation for moderate-to-severe chronic obstructive pulmonary disease: a prospective non-randomized controlled intervention study protocol
Source: Front Med (Lausanne). 2026 Jun 9;13:1861226. doi: 10.3389/fmed.2026.1861226 (PMC13287070; doi:10.3389/fmed.2026.1861226)
Supplement: Supplementary file 2 [file Table_1.docx]

Table S1. Follow-up for IoT Pulmonary Rehabilitation Group

|  | Screening/Enrollment | Follow-up Period | | | |
| --- | --- | --- | --- | --- | --- |
|  | Visit 1 | Visit 2 | Visit 3 | Visit 4 | Visit 5 |
| Study Day/Week | Day 0 | 1-month | 3-month | 6-month | 12-month |
| Informed Consent | X |  |  |  |  |
| Review Inclusion/Exclusion | X |  |  |  |  |
| Basic Information | X | X | X | X | X |
| Smoking History/Status | X | X | X | X | X |
| Other Risk Factors | X |  |  |  |  |
| Family History/Allergies | X |  |  |  |  |
| Symptom Assessment | X | X |  | X | X |
| COPD Exacerbation History | X | X | X | X | X |
| CAT Score（CAT） | X | X |  | X | X |
| mMRC Dyspnea Score（mMRC） | X | X |  | X | X |
| Physical Examination | X | X |  | X | X |
| Complete Blood Count | X |  |  | X | X |
| Pulmonary Function Tests | X |  | X | X | X |
| Six-Minute Walk Test | X |  | X | X | X |
| Medication Use | X | X | X | X | X |
| Other Treatments | X | X | X | X | X |
| Pulmonary Rehab Guidance & Eval | X | X | X | X | X |
| Adverse Event Assessment |  | X | X | X | X |
| Nutritional Assessment | X |  | X | X | X |
| Inhaler Use Assessment & Smoking Cessation Education | X | X |  | X | X |

Note: ※ Visits 3 and 5 can be conducted via telephone.

※Attempt to obtain and record CBC results during acute exacerbations in follow-up.

#Pulmonary function tests include spirometry, inspiratory muscle strength test, and bronchodilator test.

Pulmonary Rehabilitation Guidance & Assessment: Use IoT respiratory rehabilitation trainer for respiratory muscle strength training, and home monitoring of lung function and symptoms. Assess patient's usage habits and adherence to IoT management at each follow-up.

Table S2. Follow-up for Conventional Treatment Control Group

|  | Screening/Enrollment | Follow-up Period | | | |
| --- | --- | --- | --- | --- | --- |
|  | Visit 1 | Visit 2 | Visit 3 | Visit 4 | Visit 5 |
| Study Day/Week | Day 0 | 1-month | 3-month | 6-month | 12-month |
| Informed Consent | X |  |  |  |  |
| Review Inclusion/Exclusion | X |  |  |  |  |
| Basic Information | X | X | X | X | X |
| Smoking History/Status | X | X | X | X | X |
| Other Risk Factors | X |  |  |  |  |
| Family History/Allergies | X |  |  |  |  |
| Symptom Assessment | X | X |  | X | X |
| COPD Exacerbation History | X | X | X | X | X |
| CAT Score（CAT） | X | X |  | X | X |
| mMRC Dyspnea Score（mMRC） | X | X |  | X | X |
| Physical Examination | X | X |  | X | X |
| Complete Blood Count | X |  |  | X | X |
| Pulmonary Function Tests | X |  | X | X | X |
| Six-Minute Walk Test | X |  | X | X | X |
| Medication Use | X | X | X | X | X |
| Other Treatments | X | X | X | X | X |
| Pulmonary Rehab Guidance & Eval | X | X | X | X | X |
| Adverse Event Assessment |  | X | X | X | X |
| Nutritional Assessment | X |  | X | X | X |
| Inhaler Use Assessment & Smoking Cessation Education | X | X |  | X | X |

Note: ※ Visits 3 and 5 can be conducted via telephone.

※ Attempt to obtain and record CBC results during acute exacerbations in follow-up.

# Pulmonary function tests include spirometry, inspiratory muscle strength test, and bronchodilator test.
